# Nutrient pattern analysis in critically ill patients using Omics technology (NAChO) – Study protocol for a prospective observational study

**DOI:** 10.1097/MD.0000000000013937

**Published:** 2019-01-04

**Authors:** Joerg C. Schefold, Anna S. Messmer, Stefanie Wenger, Lionel Müller, Stephan von Haehling, Wolfram Doehner, Jamie S. McPhee, Michaela Fux, Kai M. Rösler, Olivier Scheidegger, Radu Olariu, Werner Z’Graggen, Serge Rezzi, Dominik Grathwohl, Tobias Konz, Jukka Takala, Bernard Cuenoud, Stephan M. Jakob

**Affiliations:** a Department of Intensive Care Medicine, Inselspital, Bern University Hospital, University of Bern, Switzerland; b Metabolic Research Unit, Department Cardiology and Pneumology, University of Göttingen, Göttingen, Germany; c Neuro Research Center, Charite University Medicine Berlin, Berlin, Germany; d Musculoskeletal Science and Sports Medicine Research Centre, Manchester Metropolitan University, Manchester, United Kingdom; e Clinical Cytomics Facility, University Institute of Clinical Chemistry and Centre of Laboratory Medicine; f Department of Neurology; g Department of Plastic Surgery; h Depts. of Neurosurgery and Neurology, Inselspital, Bern University Hospital, University of Bern; i Nestlé Research, vers-chez-les-Blanc, Lausanne; j Nestlé Health Science; k Swiss Vitamin Institute, Epalinges, Switzerland.

**Keywords:** brain injury, ICU acquired weakness, metabolic pathways, metabolomics, nutrition, proteomics, sepsis

## Abstract

**Introduction:**

Intensive care unit-acquired weakness (ICU-AW) is often observed in critically ill patients with prolonged intensive care unit (ICU) stay. We hypothesized that evolving metabolic abnormalities during prolonged ICU stay are reflected by changing nutrient patterns in blood, urine and skeletal muscle, and that these patterns differ in patients with/without ICU-AW and between patients with/without sepsis.

**Methods:**

In a prospective single-center observational trial, we aim to recruit 100 critically ill patients (ICU length of stay ≥ 5 days) with severe sepsis/septic shock (“sepsis group”, n = 50) or severe head trauma/intracerebral hemorrhage (“CNS group”, n = 50). Patients will be sub-grouped for presence or absence of ICU-AW as determined by the Medical Research Council sum score. Blood and urine samples will be collected and subjected to comprehensive nutrient analysis at different time points by targeted quantitative mass spectrometric methods. In addition, changes in muscular tissue (biopsy, when available), muscular architecture (ultrasound), electrophysiology, body composition analyses (bioimpedance, cerebral magnetic resonance imaging), along with clinical status will be assessed. Patients will be followed-up for 180 and 360 days including assessment of quality of life.

**Discussion:**

Key objective of this trial is to assess changes in nutrient pattern in blood and urine over time in critically ill patients with/without ICU-AW by using quantitative nutrient analysis techniques. Peer-reviewed published NAChO data will allow for a better understanding of metabolic changes in critically ill patients on standard liquid enteral nutrition and will likely open up new avenues for future therapeutic and nutritional interventions.

## Abstract 2

Supplemental Digital Content is available in the text

## 1 Introduction

ICU acquired weakness (ICU-AW),^[1–6]^ or critical illness myo-/poly-neuropathy (CIP/CIM/CIPM), is a key complication in critical illness and is associated with increased morbidity and mortality.^[1–3,7,8]^ Data indicate that up to 80% of ICU patients develop some form of neuromuscular dysfunction during ICU stay.^[6]^ Importantly, ICU-AW and ventilator-induced diaphragmatic dysfunction (VIDD)^[9–11]^ are associated with increased resource use, health care costs,^[1–6,12–14]^ and prolonged hospitalization.^[5–7,15]^ Although ICU-AW is a frequent complication of critical illness with important medical and economic consequences, only few data is available on evolving nutrient patterns in affected patients. While physical inactivity and impaired motor neurone and muscle fiber action potential transmission will inevitably result in loss of muscle mass, metabolic abnormalities may also contribute to the disease.^[16]^ Among others, sepsis/septic shock and/or multi-organ failure can be considered key risk factors for ICU-AW.^[3,5,6,12]^ Sepsis-induced myopathy and/or cytokine-mediated axonal neuropathy was shown to represent one underlying pathway leading to ICU-AW.^[3,17–19]^

Large-scale data on nutrient patterns in critically ill patients is currently lacking, with and without, ICU-AW. We will, therefore, study nutrient patterns over time, including amino acids, fatty acids, organic acids, lipo- and hydrosoluble vitamins, minerals, using comprehensive nutrient analysis in affected patients.^[20,21]^ In addition, we will explore the immune system/metabolic interface, for example, in tryptophan catabolism.^[22–24]^ In the current exploratory study, we will assess ICU patients with a particularly high incidence of ICU-AW (sepsis/septic shock, “sepsis group”) and patients with a presumed low ICU-AW incidence (severe head trauma and/or intracerebral hemorrhage, “CNS group”).

## 2 Objectives

### 2.1 Primary objectives

- Assessment of nutrient levels in blood over time in ICU long stayers (defined as patients with ICU length of stay ≥ 5 days) with ICU-AW (“ICU-AW group”, defined as MRC sum score < 48) vs without ICU-AW (“no-ICU-AW group”).
- Assessment of nutrient levels in blood over time in ICU long stayers (defined as patients with ICU length of stay ≥ 5 days) with severe sepsis/septic shock (“sepsis group”, according to 1992 ACCP/SCCM consensus definitions) vs without sepsis (“CNS group”) at ICU admission.

### 2.2 Secondary objectives

- Assessment of nutrient levels over time in urine in ICU long stayer (“sepsis group” and “CNS group”) with & without ICU-AW and between sepsis and CNS patients.
- Assessment of nutrient levels over time in muscle biopsy samples in all study groups (ie, “ICU-AW” vs “no-ICU-AW” and “sepsis group” vs “CNS group”), depending upon availability of a significant number of biopsies per group.

## 3 Methods

**Figure 1:**
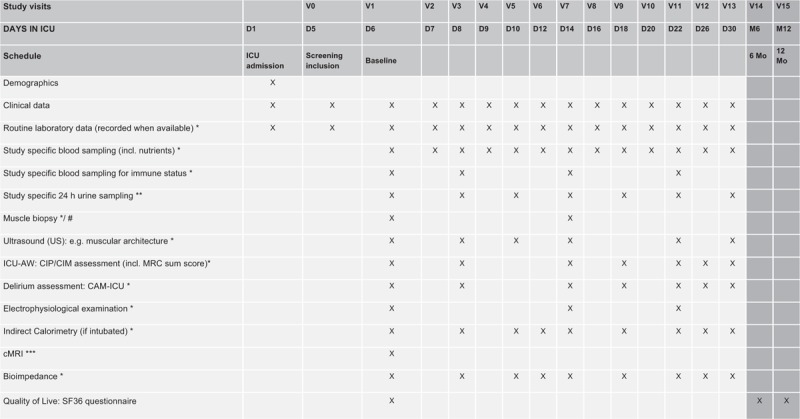
SPIRIT Figure indicating visits/study-specific assessments. ^∗^Sampling/assessment can only occur when subject hospitalized in Dept. of Intensive Care Medicine (ie, on their ICUs or IMC). ^∗∗^ Maximum target volume per study specific 24 h urine sample is 30 ml. Sampling can only occur when subject hospitalized in ICU or IMC. ^∗∗∗^ In a subgroup of patients (male, n = 15). # Depending on presence of separate consent form signed.

The “Nutrient pattern analysis in critically ill patients using Omics technology- a prospective single-center observational study” (NAChO) is a single-center prospective observational study performed in a tertiary care academic medical center department of intensive care (Department of Intensive Care Medicine, Inselspital, Bern University Hospital, University of Bern, Switzerland). Recruitment started in December 2016 (ongoing), actual protocol version is V5.0, dated March 22, 2017. Each patient attends a minimum of 1 visit (V0 [screening visit] to be performed at Day 5) and a maximum of 16 visits, depending on the date of ICU discharge (Fig. 1). A maximum of 13 visits imply study specific sampling (venous blood and/or 24 h urine). Day 1 (D1) is the day the patient is admitted to the Intensive Care Unit. Day 5 (D5) is the day patient is considered as “ICU long stayer”, which triggers V0, the screening visit. Day 6 (D6) is considered the baseline visit (V1) (Fig. 1).

Prior to study inclusion, the Investigator and/or his/her designee will explain the trial fully to the prospective patient or his/her legal representative (in case a patient is unable to provide consent). In case of willingness to participate, the patient or his/her legal representative and the Investigator or his/her designee will sign the Informed Consent Form (ICF). A separate, additional ICF will be provided for muscle biopsies. Patients can thus participate in NAChO without providing informed consent for muscle biopsies. The patient's suitability for the trial will be confirmed by the inclusion/exclusion criteria (see below). Study procedures are performed according to the planned time schedule with strict adherence to visit intervals (Fig. 1). Patients will receive trial visits (or study-specific measures) as described below, as long as they are in the ICU or Intermediate care (IMC) and informed consent is not retracted. Patients will receive standard enteral liquid nutrition, typically adapted to needs determined by indirect calorimetry. A SPIRIT checklist and Figure based on SPIRIT recommendations is attached.

### 3.1 Approvals

This study was approved by the respective competent local ethics committee on human research (Kantonale Ethikkomission, KEK, Bern Switzerland, approval number 2016–00732. Informed consent is achieved from all participants/legal representatives.

### 3.2 Population

We target to include 100 critically ill patients (n = 50 with severe sepsis/septic shock and n = 50 with severe head trauma and/or intracerebral hemorrhage of any source) with ICU length of stay ≥ 5 days.

The following inclusion criteria apply:

- Age ≥ 18 years.
- Written informed consent obtained from the patient's legal representative.
- Individuals diagnosed with severe head trauma (initial Glasgow Coma Score < 7) or intracerebral hemorrhage (“CNS group”) or severe sepsis/septic shock (“sepsis group”; defined by ACCP/SCCM consensus definitions) at any time from ICU admission until day 5 (ie, day of screening).
- Patient on the ICU for ≥ 5 days (“ICU long stayer”).

The following exclusion criteria apply:

- Language other than German or French.
- Institutionalized Individuals (eg, prisoners, severe pre-existing mental incapacity).
- Patients with known pre-existing neurological or psychiatric disease that will make informed consent likely impossible.
- Patients with known pregnancy/known to be lactating (no pregnancy testing).
- Patients on extracorporeal membrane oxygenation (ECMO).
- Clinical signs of pre-existing malnutrition (Body Mass Index < 18).
- Pre-existing evidence for history of anorexia or bulimia.
- Parenteral nutrition received within last 5 days (for more than 12 h) or evidence for parental nutrition needed within next 7 days.
- Terminal disease (ie, life expectancy < 14 days).
- Patients with active malignancy.
- Patients on biologicals interfering with metabolism (eg, infliximab).
- Patients on chronic steroid medication (ie, prednisone equivalent dose > 10 mg/day).
- Patients on neuromuscular blocking agents on a regular basis (ie, on a repetitive basis within last 48 h or evidence for need within next 7 days).
- At screening (day 5): prescribed caloric intake substantially higher (ie, > 150% of caloric need of indirect calorimetry) than indirect calorimetric results (in intubated patients) or that is, standard-of-care (25 Kcal/kg body weight/day).
- Severe adipositas (Body Mass Index > 40).
- Known pre-existing allergy to standard enteral formulae.
- Patient previously enrolled to an interventional trial that is not considered standard-of-care (eg, investigation of new medication/drug or medical devices).

### 3.3 Patient discontinuation criteria

A patient may be discontinued prematurely for the following reasons (patients who discontinue before completing the trial will not be replaced):

- Patient requests to leave the trial.
- If, in the Principal Investigator's opinion, continuation in the trial would be detrimental to the patients’ well-being.
- Sponsor or Independent Ethics Committee (EC)/Institutional Review Board (IRB) decides to terminate trial for significant reasons.

### 3.4 Outcome measures

#### 3.4.1 Primary endpoints

- Differences in blood (plasma/serum) nutrient levels over time in patients with/without ICU-AW (ie, “ICU-AW” vs “no-ICU-AW” groups. Primary comparison of interest is between day 6 (baseline visit) and day 14.
- Differences in blood (plasma/serum) nutrient levels over time in patients in “sepsis group” vs “CNS group”. Primary comparison of interest is between days 6 (baseline visit) and 14.

#### 3.4.2 Secondary endpoints

- Differences in urine nutrient levels over time in patients with/without ICU-AW (ie, “ICU-AW” vs “no-ICU-AW” groups. Primary comparison of interest is between days 6 (baseline visit) and 14.
- Differences in urine nutrient levels over time in patients in “sepsis group” vs “CNS group”. Primary comparison of interest is between days 6 (baseline visit) and 14.
- Subgroup analysis: differences in nutrient levels at days 6 (baseline visit) and 14 in muscle biopsy samples (if available) of ICU long stayer patients in study groups (ie, “ICU-AW” vs “no-ICU-AW” and “sepsis group” vs “CNS group”).

#### 3.4.3 Exploratory endpoints

Please refer to supplemental content.

### 3.5 Sample collection and sample handling

Blood samples are collected in the morning and immediately stored at the recommended temperature (usually on ice). Blood collection tubes, intermediate tubes and sample vials remain at the recommended temperature during pre-analytical sample handling (usually on ice). Sample aliquots for vitamin C and liposoluble vitamins are prepared using specific antioxidants to avoid oxidation of the vitamins. All aliquots are stored at −80°C until the start of analyses. For collection of 24 h urine samples, the urine is collect in standard of care urine collectors. Urine collectors are then completely discharged in pre-cleaned plastic containers, sealed and homogenized by gently shaking. Subsequently, the specific density and total weight of the urine are determined before aliquots for analysis are collected and stored at −80°C. Muscle biopsies (open surgical biopsy from Musculus vastus lateralis) will be handled according to routine pre-analytical requirements for histological staining and nutrient analysis.

### 3.6 Study specific analysis and clinical measures

#### 3.6.1 Laboratory analyses

In brief, we aim to quantify the following levels of nutrients by using mass spectrometry and clinical chemistry platforms (please also refer to supplemental content)^[25,26]^: Blood (plasma/serum): fatty acids, amino acids and metabolites, organic acids, electrolytes/minerals, vitamins, routine clinical markers (phosphate, albumin, pre-albumin, total protein, alanine aminotransferase, total bilirubin, creatinine kinase, alkaline phosphatase, creatinine, urea, lipase, cholesterol, C-reactive protein/inflammatory markers, ferritin, total IgG, cortisol, ceruloplasmin, transferrin, parathyroid hormone and exploratory markers of muscle metabolism.

Urine: specific density, electrolytes/minerals, creatinine, uric acid, inflammatory markers.

Muscle: should circulating nutrient signatures associated with ICU-AW be identified, complementary analysis of intramuscular levels of specific nutrients (eg, minerals, amino acids) will be performed, depending on sample availability.

#### 3.6.2 Imaging: ultrasound, cMRI

Ultrasound (US) is a bedside tool used in this study to assess skeletal muscle architecture and muscle wasting in ICU patients.^[27]^ Proximal muscles of the lower extremities are particularly prone to muscle wasting.^[28]^ The Musculus vastus lateralis (VL) is easily accessible and allows reliable and reproducible recording of muscle thickness. US assessments will be performed using a 7.5 Mhz linear probe with patients in the supine position with both legs extended. Whenever possible, the right leg will be investigated at 3/5 of the distance between anterior superior iliac spine and the lateral superior patellar border (longitudinal axis, perpendicular to skin). The following US parameters will be assessed: VL muscle thickness and thickness of subcutaneous tissue (both in long axis, edge to edge). Image intensity, fascicle length and pennation angle will be available in secondary analyses. Using cMRI imaging, tensor-based morphometry will be assessed to investigate structural changes in brain morphology.

#### 3.6.3 Bioimpedance

Bioimpedance (BIA corpus RX 4004, Medi-Cal, Karlsruhe, Germany) will be used for body composition analysis. The following indices will be assessed: basal metabolic rate (BMR), extracellular mass (ECM), body cell mass (BCM), fat mass (FM), fat free mass (FFM), total body water (TBW), extracellular water (ECW), intracellular water (ICW), water balance, phase angle (PA), vectorial data (reactance, resistance).

#### 3.6.4 Clinical assessments: ICU-AW, delirium, indirect calorimetry

ICU-AW will be assessed clinically as determined by the Medical Research Council (MRC) sum score (ICU-AW defined as MRC sum score < 48). Delirium will be assessed by using the Confusion Assessment Method for the Intensive Care Unit (CAM-ICU).^[29]^ Indirect calorimetry will be performed in cases patients are intubated using standard mechanical ventilators (Servo-I, Maquet, Getinge, Sweden).

#### 3.6.5 Electrophysiological examination (ENMG)

Electrophysiologic examinations (conventional nerve conduction studies, conventional electromyography) will be performed. For assessment of critical illness polyneuropathy (CIP), occurrence of axonal motor and sensory polyneuropathy will be used for diagnosis. Absence of a decremental response on repetitive nerve stimulation will be checked for to rule out concurrent neuromuscular junction dysfunction. The following anatomical sites will be assessed: Nn. peroneus, suralis, medianus. For assessment of critical illness myopathy (CIM), additional EMG and direct muscle stimulation (and calculation of the compound muscle action potential (CMAP) ratio nerve vs muscle stimulation) will be performed from the M. tibialis anterior. CMAP amplitudes less than 80% of the lower limit of normal in 2 or more nerves without conduction block and sensory nerve action potential amplitudes more than 80% of the lower limit of normal will be considered relevant for diagnosis of CIM in parallel to typical findings in EMG and a pathological nerve/muscle CMAP ratio. In all investigations, the right extremity will preferentially be investigated.

### 3.7 Data collection and management

Collected data will be entered in the database via electronic case report files (eCRF). Web-based access to the database is provided on a 24/7 basis. Data will be encoded by using a unique patient identification number. Data will be handled according to the requirements of local data protection authorities and respective ethical committees. All data will be stored in a protected environment for a minimum of 10 years and anonymized if requested by the authorities. The following clinical data will be collected in NAChO: age, gender, ethnicity, major preexisting comorbidities and medication, clinical scores (Acute Physiology and Chronic Health Evaluation II, Simplified Acute Physiology Score II, Sepsis-related Organ Failure Assessment score), date of hospital and ICU admission, routine laboratory data, days on any renal replacement therapy (RRT), days on mechanical ventilation, days on catecholamines, cumulative dose of muscle relaxants, cumulative dose of analgosedatives, cumulative dose of catecholamines, cumulative steroid dose.

The clinical database developed for this study comply with the Good Clinical Practices (GCP) predicate rule requirements, laws and regulations (Personal data protection) and allows an audit of actions performed by users. All data modifications will be documented in an audit trail file. After the last verification, the relevant data will be locked. The system is compliant with e-signature laws (particularly the Title 21 CFR Part 11 of the Code of Federal Regulations). All eCRF data will be validated by a Clinical Data Manager. Computerized edit checks programmed to validate the data will be performed with data discrepancies triggering automatic queries.

### 3.8 Trial monitoring

Trial monitoring is performed by the Clinical Trials Unit (CTU) of the University of Bern, Switzerland. Monitoring of trial data and documentation ensures that protocol requirements, applicable local laws, ICH guidelines, and Investigator obligations are fulfilled.

### 3.9 Safety

Due to the observational nature of the trial, adverse events (AEs) will be reported only for unusual undesired medical events that seem unrelated or atypical to the underlying critical disease. As critically ill patients may experience potential life threatening events by definition, severe adverse events (SAEs) will be reported in cases of death on the ICU only. SAEs related to study procedures will be reported also.

### 3.10 Statistical analysis

Baseline considerations: Patients entering the study at day 5 are considered ICU long stayers. It is expected to have around 50 to 60% of the patients still at the ICU at day 14. The main objective is to characterize the change from baseline (defined as day 5) for nutrient levels in blood of patients with and without ICU-AW and in “sepsis group” vs “CNS group”. Primary characterization of interest is change of nutrient levels in blood (plasma/serum) from baseline (day 6) to day 14 (visit 7). Characterization of the change from baseline (day 6) of nutrient levels in blood (plasma/serum) over the ensuing days will be of supportive interest. Secondary objectives are to characterize the change of nutrient levels in muscle and in urine from baseline to day 14, day 22 and day 30 in patients with vs without ICU-AW and in “sepsis group” vs “CNS group”.

All statistical analyses will be performed using either SAS 9.3 (or higher) or R 3.0 (or higher). Baseline characteristics will be described for all patients on the full analysis set. Summary statistics for primary and secondary endpoints will be described for all patients on the full analysis set at baseline and at each post baseline visit. Continuous variables will be summarized using the following descriptive statistics:

- For approximately normally distributed variable, the number of non-missing values, the arithmetic mean, the arithmetic standard deviation, the minimum and the maximum values.
- For approximately log-normally distributed variables, the number of non-missing values, the geometric mean, the geometric standard deviation, the minimum and the maximum values.
- For non-normally and non-log-normally distributed variable, the number of non-missing values, the median, the 25th and 75th percentiles, the minimum and the maximum values.

Categorical variables will be summarized using frequencies and percentages (out of total number of non-missing values). Unless otherwise specified, each test will be performed using a significance level of 5%. Each model based statistic will be presented both with the associated 2-sided 95% confidence interval and two-sided *P* value. No multiplicity adjustment will be considered. Based on current results, data will be controlled for quality. Extreme outliers will be detected based on inter-quartile range (IQR) detection. Based on the proportion of missing values, variables will be considered for inclusion or exclusion from subsequent analysis.

#### 3.10.1 Primary analyses

For each nutrient level in blood (plasma/serum) of ICU long stayer patients, the change from baseline to day 14 (visit 7) will first be analyzed using a linear model, adjusting (but not restricted to) for nutrient level at day 6 and ICU admission condition (“sepsis group” or “CNS group”) aiming to evaluate the difference between ICU long stayers in sepsis and ICU long stayers in the CNS group. In a second model, change from baseline for nutrient level at day 14 will be analyzed using a linear model, adjusting (but not restricted to) for nutrient level at day 6 and the ICU-AW status aiming in evaluating the difference between patient with and without ICU-AW. Note that these analyses of the change from baseline for nutrient levels in blood may be combined with the change from baseline in nutrient levels in blood observed at other relevant subsequent visit using a linear mixed models or an ANOVA for repeated measures approach. In case of severe deviation from the normality hypothesis, log transformation or Box-Cox transformation may be applied. Interaction between ICU admission condition and ICU-AW status may be considered in these linear models as an explanatory analyses. The following SAS code may be used when change from baseline at day 14 is analyzed alone: PROC MIXED DATA = ICU; CLASS cond; MODEL CHG = BASE cond /S; RUN; where cond is the variable indicating the condition of the ICU patient, BASE is the baseline measurement, CHG the change from baseline and ICU is the dataset containing all these variables.

As the number of patients in the CNS group who will die or be discharged from ICU may be different from the number of patients in the sepsis group, a competing risk analysis on these 2 populations will be performed as supportive analyses. If the risks for both patient populations are similar, we may have more confidence in any finding of the ANCOVA model. The initial states are patients at day 6 in “sepsis group” or “CNS group”. The cumulative incidence function will be calculated for ICU-AW and for ICU discharge or death (no distinction between discharge and death). The Aalen-Johansen estimator will be used. A proportional sub-distribution hazard model according to Fine and Gray^[30]^ on the event of interest ICU-AW, stratified by diagnosis (sepsis or CNS at ICU admission) with variable selection by boosting as previously proposed.^[31]^ Variables which undergo boosting are the ones of the nutrient profile measured at day 6 (first competing risk analysis) and changes from baseline at day 14 for the nutrient profile (second competing risk analysis). Boosting will preferably be performed with the R package CoxBoost available on the CRAN.

#### 3.10.2 Secondary analyses

Similar statistical approaches to the primary analyses will be used to evaluate nutritional and metabolic signatures in urine and muscle (if available) of ICU-long stayers:

- The change from baseline to day 14 will be analyzed using a linear model, adjusting (but not restricted to) for nutrient level at day 6 and ICU admission condition (“sepsis group” or “CNS group”);
- The change from baseline to day 14 will be analyzed using a linear model, adjusting (but not restricted to) for nutrient level at day 6 and the ICU-AW status; Note that these analyses of the change from baseline for nutrient levels in muscle and urine may be combined with the change from baseline in nutrient levels observed at other relevant subsequent visit using in linear mixed models or an ANOVA for repeated measures approach. In case of severe deviation from the normality hypothesis, log transformation or Box-Cox transformation may be applied. Interaction between ICU admission condition and ICU-AW status may be considered in these linear models as an additional explanatory variable.

#### 3.10.3 Exploratory analyses

Exploratory analyses will be finalized in the Statistical Analysis Plan.

### 3.11 Power analysis

This is a prospective observational trial with 50 patients with severe sepsis/septic shock (“sepsis group”) with an expected high incidence of ICU-AW and 50 patients (“CNS group”) with an expected low incidence of ICU-AW. Due to the observational non-interventional and exploratory nature (estimation of effect size is the focal point) of the study, a formal power calculation was refrained from as it was not deemed useful.

### 3.12 Timeline

2016–2017: Finalization of research strategy, finalization of protocol, approvals

2017–2019: First patient in, estimated end of trial in 2019

Q3-Q4 2019: Analysis of trial results and publication

### 3.13 Publication/authorships

Before publication of the trial results in an academic journal, a publication committee will be formed which will include members of the involved parties and which will be chaired by the principle investigator. A main publication (eg, termed “Nutrient pattern Analysis in critically ill patients using Omics technology (NAChO)- a single-center prospective observational trial”) will aim to summarize key clinical and laboratory findings as well as nutrient patterns. In this main publication, the first and corresponding author will be the Principle investigator and the last author will be another member of the clinical team. Co-Authorship will be granted on the basis of personal input in accordance to Vancouver definitions. If applicable, additional publications (eg, on MRI results, electrophysiological findings, and/or detailed course of specific nutrient or lab markers/patterns) will include members of all involved parties and authorship will be granted on the basis of personal input in accordance to Vancouver definitions.

## 4 Discussion

Previous data indicate that intensive care unit (ICU)- acquired weakness (ICU-AW) significantly affects morbidity and mortality rates of affected critically ill patients.^[2,3,6]^ However, metabolic changes in respective patients and how sepsis may influence them is largely unknown. We thus investigate underlying nutritional and muscular patterns in septic and non-septic long staying ICU patients with/without ICU-AW that receive enteral nutrition using a standard liquid enteral formula. Using comprehensive nutrient analysis, we will assess changes in nutrient patterns in blood (plasma/serum), urine, and muscular tissue (if available) over time in critically ill patients with the aim to identify ICU-AW-specific nutrient patterns. NAChO data and respective computational models will allow for a better and more detailed understanding of metabolic changes in critically ill patients and will likely open up new avenues for future therapeutic and nutritional interventions.

### 4.1 Strengths and limitations

Comprehensive assessment of nutrients and metabolic indices, including amino acids, fatty acids, organic acids, vitamins, minerals, routine clinical parameters, immune function markers including analysis of immune system/metabolic interface, in distinct biological matrices (blood plasma/serum, urine, muscle) was previously not performed in critically ill patients. In the light of the specific patient population included, the sample size may be considered large. Following comprehensive analysis, we will be able to identify novel metabolic and nutrient patterns using sophisticated computational models. The exploratory nature of the trial will allow for hypothesis driving in multiple areas of nutritional/metabolic research, and this is regarded as major strength.

A number of limitations apply that deserve discussion. First, this is a single-center trial with a somewhat limited number (n = 100) of critically ill patients. Although this may be the first larger analysis to generate comprehensive nutrient pattern data in critically ill patients, and following expert statistical consultation, a formal sample size calculation was deliberately refrained from. The focal point of the current exploratory observational trial is estimation of effect sizes. Second, collection of samples was deliberately chosen to start late, that is, in ICU patients with prolonged length of stay (“ICU long stayers”). This was performed as respective ICU long stayers are the population of critically ill patients with a particular high morbidity and mortality, high resource use, and a high incidence of ICU-AW. It is speculated that this population may benefit most from respective therapeutic interventions. Third, as in all observational studies, underlying molecular mechanism can only be concluded on in further studies, for example, from muscle tissues. Thus, associations and patterns rather than molecular mechanisms will be explored. Fourth, only patients with CNS disorders and/or sepsis are included, precluding a formal control group. However, we deliberately choose to include a group of critically ill patients with a presumed high incidence (“sepsis group”) and low incidence (“CNS group”) of ICU-AW. Finally, the amount of liquid enteral nutrition delivered to each individual patient might not always be the same and might theoretically impact the results across groups. Nevertheless, the amount of enteral nutrition/caloric intake will typically be adapted to the results of indirect calorimetric measurements. Importantly, the chosen enteral liquid nutritional intake regimen may best reflect clinical reality rather than any artificial trial conditions.

### 4.2 Trial status

The trial is currently actively recruiting patients. Completion of patient recruitment is expected for Q1-Q2 of 2019.

## Acknowledgments

The authors thank all trial physicians, nurses, research nurses, data managers, laboratory and statistical staff for their dedicated support of NAChO.

## Author contributions

JCS drafted and finalized the protocol/manuscript, applied for EC approval, initiated the study, recruited patients, and supervised the trial. ASM, SW, LM, MF, KR, and WZG helped to draft the manuscript, performed study assessments, recruited patients, and revised the manuscript for important intellectual content. WZG, SvH, WD, and JMP provided expert consultation, developed the ultrasound research (JMP) and partly the metabolic research strategy (SvH, WD), and revised the study protocol for important intellectual content. OS and RU performed study measures, and drafted the manuscript for important intellectual content. DG provided expert statistical consultation and drafted the statistical analysis plan. TK contributed to the drafting of the protocol and revised the manuscript. SR, BC, JCS, SMJ and JT developed the research strategy and clinical design, drafted the protocol and revised the manuscript. All authors read and approved the final version of the manuscript.

**Conceptualization:** Joerg C. Schefold, Stephan von Haehling, Serge Rezzi, Jukka Takala, Bernard Cuenoud, Stephan M. Jakob.

**Formal analysis:** Dominik Grathwohl.

**Investigation:** Anna Sarah Messmer, Stefanie Wenger, Lionel Müller, Olivier Scheidegger, Radu Olariu.

**Methodology:** Joerg C. Schefold, Stephan von Haehling, Wolfram Doehner, Jamie S. McPhee, Michaela Fux, Kai M. Rösler, Olivier Scheidegger, Radu Olariu, Werner Z Graggen, Serge Rezzi, Jukka Takala, Bernard Cuenoud, Stephan M. Jakob.

**Project administration:** Joerg C. Schefold.

**Writing – original draft:** Joerg C. Schefold, Radu Olariu, Serge Rezzi, Dominik Grathwohl, Tobias Konz, Jukka Takala, Bernard Cuenoud, Stephan M. Jakob.

**Writing – review & editing:** Joerg C. Schefold, Anna Sarah Messmer, Stefanie Wenger, Lionel Müller, Stephan von Haehling, Wolfram Doehner, Jamie S. McPhee, Michaela Fux, Kai M. Rösler, Olivier Scheidegger, Radu Olariu, Werner Z Graggen, Serge Rezzi, Dominik Grathwohl, Tobias Konz, Jukka Takala, Bernard Cuenoud, Stephan M. Jakob.

## Supplementary Material

Supplemental Digital Content
