# Supplementary material for: Nutrient pattern analysis in critically ill patients using Omics technology (NAChO) – Study protocol for a prospective observational study
Source: Medicine (Baltimore). 2019 Jan 4;98(1):e13937. doi: 10.1097/MD.0000000000013937 (PMC6344160; doi:10.1097/MD.0000000000013937)
Supplement: Supplemental Digital Content [file medi-98-e13937-s001.pdf]

Nutrient pattern analysis in critically ill patients using Omics technology (NAChO) - study protocol for a prospective observational study

Joerg C. Schefold, Anna S. Messmer, Stefanie Wenger, Lionel Müller, Stephan von Haehling, Wolfram Doehner, Jamie S. McPhee, Michaela Fux, Kai Rösler, Olivier Scheidegger, Radu Olariu, Werner Z'Graggen, Serge Rezzi, Dominik Grathwohl, Tobias Konz, Jukka Takala, Bernard Cuenoud, Stephan M. Jakob

<u>Methods and analysis section</u>

Exploratory endpoints in NAChO

- Correlation of muscle physiology markers with nutrient levels in blood (plasma/ serum) and urine in “ICU-AW” vs. “no-ICU-AW” groups.
- Molecular mechanisms associated with ICU-AW or sepsis in muscle at transcriptomic and proteomic levels, if number of collected tissue samples allows.
- Descriptive assessment of clinical/ laboratory course, evolution of electrophysiological indices, and course of disease severity (incl. scores) related to levels of specific nutritional parameters/ nutritional profiles.
- Course of key muscular ultrasonography indices before background of respective study groups (“ICU-AW” vs. “no-ICU-AW” and “sepsis group” vs. “CNS group”).
- Course of ICU-AW (incl. severity scores) over time in respective study groups (i.e. ”ICU-AW group” vs. “no-ICU-AW group” and “sepsis group” vs. “CNS group”).
- Course of electrophysiological indices over time in respective study groups (i.e. “ICU-AW” vs. “no-ICU-AW” and “sepsis group” vs. “CNS group”).
- Course of bio-impedance indices over time in respective study groups (i.e. “ICU-AW” vs. “no-ICU-AW” and “sepsis group” vs. “CNS group”).
- Descriptive analysis of evolution of quality of life, and clinical outcome over time in respective study groups (i.e. “ICU-AW” vs. “no-ICU-AW” and “sepsis group” vs. “CNS group”).
- Tensor based morphometry as assessed by cerebral magnetic resonance imaging (cMRI), descriptive analysis.

Study specific analysis and clinical measures

<u>Laboratory analyses</u>: Using mass spectrometry, we aim to assess the following metabolic indices:

<u>Fatty acids</u>: butyric C4:0, caproic C6:0, caprilic C8:0,capric C10:0, undecanoic C11:0, lauric C12:0, tridecanoic C13:0, myristic C14:0, pentadecanoic C15:0, palmitic C16:0, heptadecanoic C17:0, stearic C18:0, arachidic C20:0, heneicosanoic C21:0, behenic C22:0, lignoceric C24:0, myristoleic C14:1 n-5, cis-10-pentadecenoic C15:1 n-5, palmitoleic C16:1 n-7, cis-10-heptadecenoic C17:1 n-7, elaidic C18:1 n-9 trans, oleic C18:1 n-9 cis, cis-11-eicosenoic C20:1 n-9, erucic C22:1 n-9, nervonic C24:1 n-9, linoelaidic C18:2 n-6 trans, linoleic C18:2 n-6 cis, gamma-linolenic C18:3 n-6, alpha-linolenic C18:3 n-3, cis-11,14-eicosadienoic C20:2 n-6, cis-8,11,14-eicosatrienoic C20:3 n-6, cis-11,14,17-eicosatrienoic 20:3 n-3, arachidonic C20:4 n-6, cis-13,16-docosadienoic 22:2 n-6, cis-5,8,11,14,17-eicosapentanoic (EPA) C20:5 n-3, cis-4,7,10,13,16,19-docosahexaenoic (DHA) C22:6 n-3

Amino acids: alanine, β-alanine, sarcosine, arginine, monomethylarginine, assymetric dimethylarginine, symmetric dimethylarginine, asparagine, aspartic acid, citrulline, glutamic acid, glutamine, glycine, histidine, 1-methylhistidine, 3-methylhistidine, isoleucine, leucine, lysine, methionine, ornithine, phenylalanine, proline, serine, taurine, threonine, tryptophan, tyrosine, valine, hydroxyproline, ethanolamine, α-aminobutyric acid, β-aminoisobutyric acid, γ-aminobutyric acid, cystein, homocystein

Organic acids: 2-ketobuyric acid, 3-methyl-2-oxobutyric acid, 3-methyl-2-oxopentanoic acid, 4-methyl-2-oxopentanoic acid

Minerals: boron, magnesium, aluminium, phosphate, sulfur, potassium, calcium, titan, vanadium, chrome, mangan, iron, cobalt, nickel, copper, zinc, arsen, selenium, bromide, rubidium, strontium, molybdenum, cadmium, tin, iodine, cesium, barium, mercury, lead, sodium, chloride

Vitamins: thiamine, riboflavin, pantothenic acid, pyridoxal, pyridoxine, pyridoxamine, pyridoxal phosphat, pyridoxic acid, biotin, folic acid, methyl tetrahydrofolic acid, ascorbic acid, and liposoluble vitamins.

Clinical chemistry: phosphate, albumin, total protein, alanin-aminotransferase, total bilirubin, creatine kinase, alkaline phosphatase, creatinine, urea, lipase, high sensitivity C-reactive protein, total cholesterol, interleukin 6, interleukin 10, soluble tumor necrosis factor receptor 1, ferritine, total immunoglobulin G, pre-albumin, cortisol, ceruloplasmin, transferrin, parathyroid hormone.
